# Low dose clomiphene citrate as a mild stimulation protocol in women with unsuspected poor in vitro *fertilization* result can generate more oocytes with optimal cumulative pregnancy rate

**DOI:** 10.1186/s13048-018-0408-x

**Published:** 2018-05-04

**Authors:** Humphrey Ochin, Xiang Ma, Lin Wang, Xuan Li, Jie Song, Yan Meng, Jiandong Shen, Yu-Gui Cui, Jiayin Liu

**Affiliations:** Clinical Center for Reproductive Medicine, 300 Guangzhou Road. First Affiliated Hospital of Nanjing Medical University, 210029, The State Key Laboratory of Reproductive Medicine., Nanjing, People’s Republic of China

**Keywords:** Clomiphene citrate, Long GnRH-a protocol, Mild ovarian stimulation, Unsuspected poor IVF results

## Abstract

**Background:**

The use of long protocol during controlled ovarian stimulation for assisted reproduction attracts high dosage of gonadotropins. High dose of gonadotropins can be detrimental to oocyte development, which affects its quality and compromises the treatment outcome. Mild stimulation protocols that attract low dose gonadotropins could be useful alternative regimen to address such problems. This study evaluated the efficacy of low dose clomiphene citrate based protocol plus low dose gonadotropins on predicted normal responder patients who had unsuspected poor in vitro fertilization (IVF) result, following an initial stimulation with long gonadotropin-releasing hormone (GnRH) agonist protocol.

**Methods:**

This a retrospective study of 65 infertile women who underwent 130 cycles in our center from January 2011 to December 2014. The initial IVF cycle (Group 1) was treated with long GnRH-a protocol plus a high dose of gonadotropins (≥150 IU/d), while second IVF cycle (Group 2) had low dose clomiphene citrate based protocol plus low dose gonadotropins (75–112.5 IU/d).

**Results:**

The rate of cumulative pregnancy/started cycle (9.2% [6/65] vs. 51% [33/65]; *P* < 0001) was significantly better in CC protocol than the long GnRH agonist protocol. The number of oocytes retrieved was also higher in CC protocol compared to the long protocol (7.26 ± 1.95 vs. 5.98 ± 1.31; *P* = 0.03). There was a lower number of patients without embryos (12.31% vs. 33.85%; *p* < 0.0001) in CC protocol than long protocol.

**Conclusions:**

This study showed a better cumulative pregnancy rate in the low dose CC based protocol. There was a higher number of oocytes retrieved after using a lower total dose of recombinant FSH in CC protocol. Thus, clomiphene treatment plus low dose rFSH can be an alternative option for such patients in second cycle stimulation instead of repeating long protocol regimen. Randomized controlled studies with larger number of patients will be needed for more accurate evidence.

## Background

The prediction of ovarian response prior to IVF treatment using the age, antral follicle count (AFC), anti-mullerian hormone (AMH), basal FSH and E_2_ levels still underperform during conventional controlled ovarian stimulation in some patients with predicted normal ovarian response, thereby yielding an unsuspected poor IVF outcome. This could be as a result of poor quality oocytes with poor quality embryos, possibly inflicted by the stimulating protocol [1]. Several stimulation protocol strategies such as microflare lupron in the follicular phase, estradiol in the luteal phase, oral medications (clomiphene or letrozole) prior to initiation of/start with exogenous gonadotropins had been employed to improve the result of such patients. Those alternative protocols are mainly applied after two or three times IVF treatment failure following long protocol stimulation [2], which attracts more expenses and time-consuming.

However, a long protocol with the high dose of gonadotropins usually recruits many follicles to generate enough number of oocytes and embryos to increase chances of pregnancy but does not necessarily increase the qualities of oocytes and embryos. Mild stimulation approach has been reported of yielding quality oocytes and embryos from few recruited follicles available in ovaries at risks without compromising pregnancy results [3]. Controlled ovarian stimulation is assessed based on pregnancy and live birth rate achieved, and these usually depend on the quality of oocytes, embryos obtained, stimulation approach and the endometrium thickness. Therefore, choice of choosing stimulation approach should be based on protocols with lower side effects on the oocyte quality without decreasing the IVF results instead of repeating protocols suspected to have a more adverse effect after an initial stimulation. Mild stimulation (MS) regimen reduces patient’s physical and emotional burden [3] because it is associated with low ovarian hyperstimulation syndrome (OHSS) and production of quality oocytes and embryos without affecting pregnancy results. MS protocol was first proposed by Zarek and Muasher [4], as a deviation from standard stimulation protocol which provides a more convenient and low cost effective to patients.

In 2007, mild stimulation was recognized by International Society for Mild Approaches in Assisted Reproduction (ISMAAR) association as an alternative to conventional stimulation. The ISMAAR defined mild stimulation as administration of gonadotropins in a low dose and/or shorter duration, or use of oral compounds (such as anti-estrogen or aromatase inhibitors) either alone or in combination with gonadotropins and antagonist as co-treatment, aiming at collecting less than eight oocytes [5, 6]. Clomiphene citrate protocol mimics natural cycle, exhibits lower potential risks, less expensive, encourages fewer visits for the patient and fewer burdens during the IVF treatment [7, 8]. In this study, we intend to evaluate the efficacy of low dose clomiphene citrate based protocol plus low dose gonadotropins among unsuspected poor in vitro fertilization (IVF) results from predicted normal responder patients initially treated with long gonadotropin-releasing hormone (GnRH) agonist.

## Methods

### Patients

This is a retrospective study of infertile women with predicted normal ovarian response in our reproductive center from January 2011 to December 2014, involving 65 self-compared patients who underwent 130 cycles of IVF treatment using two different protocols. We included patients with predicted normal ovarian response that had long GnRH agonist protocol in their first IVF cycle and had low dose clomiphene citrate based protocol plus low dose gonadotropins (as rFSH) in their second IVF cycle. Patients have baseline characteristics of age range of 22–38 years, basal FSH < 10 IU/L, E_2_ <70 pg/mL, AFC > 5 follicles, and body mass index (BMI) <26 kg/m^2^. Patients were considered predicted normal responder based on their baseline characteristics and having greater than 5 oocytes retrieved from the previous cycle, but with poor IVF results. Ethical approval was obtained from the institutional review board (IRB) committee of the first affiliated hospital for Nanjing medical university before the collection of data for analysis (IRB reference no. 2016-SR-216).

### Controlled ovarian hyperstimulation

Patients in the first IVF cycle was given long GnRH agonist (Suprefact, Hoechst, Germany) of 0.05–0.1 mg/d subcutaneously, started from the luteal phase of the previous menstrual cycle until 14 days into the next cycle to achieve pituitary down-regulation. Avoidance of desensitization was achieved by checking serum E_2_ level to be < 50 pg/ml, luteinizing hormone (LH) < 5 IU/L and making sure there is antral follicular diameter < 8 mm with the aid of transvaginal ultrasound. Recombinant FSH ≥150 IU/day (GONAL-f®, Merck Serono, Switzerland) was administered until the day of maturation triggering with human chorionic gonadotropin (hCG) injection.

In the second IVF cycle of this study, patients were stimulated with CC (Serophene, Merck-Serono, Switzerland) of 50 mg/d orally, plus rFSH (GONAL-f®, Merck Serono, Switzerland) of 75~ 112.5 IU/d subcutaneously, started from day 3 of menstrual cycle until a day before hCG injection. In this cycle, the same patients from the previous cycle were still stimulated for IVF treatment due to their cycle failures or the need for another child. A serial monitoring of both cycles with transvaginal ultrasound and/or hormonal assay was adopted to monitor follicle growth every 2–3 days depending on the rate of follicle size growth per day starting from day 7–9 of the stimulating cycle. When the diameter of a dominant follicle was ≥18 mm or 3 follicles = 17 mm, or 4 follicles = 16 mm with serum level of E_2_ ≥ 1100 pmol/L per follicle, maturation triggering was achieved by injecting 6500 IU of hCG (Profasi, Serono Pharma) subcutaneously. Oocyte retrieval was done 36 h after maturation triggering under ultrasound guidance.

### IVF/ICSI treatment

Standard insemination: in vitro fertilization (IVF) or intracytoplasmic sperm injection (ICSI) was performed as clinically appropriate. Embryos were cultured in sequential Growth medium (Sage, Englewood, CO) and incubated in 6% CO_2_, 5% O_2_, and 89% N_2_, then scored using evidence-based 1–4 level integrated morphology cleavage (IMC) embryo score according to Holte et al. [9]. All embryo transfer (fresh ET in the first cycle and frozen-thawed ET in the second cycle) was done using a soft catheter (Sydney, Cook, Australia) under a guided abdominal ultrasound. Serum level of HCG (>200 IU/mL) measured two weeks after embryo transfer (ET) and if positive, clinical pregnancies were confirmed by transvaginal ultrasound examination; observing the presence of intrauterine gestational sac with fetal cardiac activity 14 weeks after ET. The luteal phase was supported either with 400 mg/d of progesterone and 20 mg/d of dydrogesterone or 90 mg/d of Crinone and 20 mg/d of dydrogesterone, started from the day of oocyte pickup until 8–10 weeks of pregnancy from the day of ET.

### Outcome measures

The primary measure is cumulative pregnancy rate, while secondary measures include the number of oocytes retrieved, fertilization rate, number of patients without embryos, average number of transferred embryos and total dose of gonadotropins used.

### Statistical analysis

Results are presented either as the mean ± standard deviation (SD), percentages or absolute numbers. The continuous variables (e.g., Age, and AFC) were expressed as mean ± SD. whereas categorical variables (e.g., clinical outcomes and type of infertility) were presented as percentage (%) and absolute numbers (n). Patient age, Infertility duration, number of retrieved oocytes, as well as fertilization rate and the average number of transferred embryos were the main variables analyzed against cumulative pregnancy rate in this study. The variables and the cumulative pregnancy rate were calculated by using GraphPad Prism (GraphPad Inc., 2007) 5.01, software, to estimate the expected CPR. The Column analysis (t-test and nonparametric tests) was used, which is not based on a specific assumption with respect to the distribution of the data. Between-group differences were tested by the paired t-test in normality test and by Mann-Whitney U test in a nonparametric test. In order to assess homogeneity within various groups in this study, the paired t-test or Mann-Whitney U test significance was noted when *P* < 0.05. Cut-off values for age, infertility duration, etc. were determined by the regression.

## Results

We analyzed 65 self-compared infertile women who underwent IVF cycles in two different protocols. All the baseline characteristics were similar in both groups (Table 1). There was a significant difference in cumulative pregnancy rate (9.2% [6/65] vs. 51% [33/65]; *p* < 0.0001) when compared in long GnRH agonist protocol versus CC protocol (Table 2). The number of retrieved oocytes (5.98 ± 1.31 vs. 7.26 ± 1.95; *P* = 0.03), total dose of rFSH (1719.41 ± 545.199 IU vs. 981.54 ± 236.976 IU; *P* < 0.0001) used, and endometrium thickness (10.40 ± 2.36 vs. 7.26 ± 2.19; *P* < 0.0001) were significantly different in long GnRH agonist protocol when compared to CC protocol respectively (Table 3). Few patients without embryos (12.31% vs. 33.85%; *p* < 0.0001) was found in CC protocol than long protocol.

**Table 1:** Baseline characteristics of the patients

Mean ± SD; %	Long GnRHa Protocol (*n* = 65)	CC Protocol (*n* = 65)	*P-*Value	Mean difference (95% Confidence Interval)
Age(years)	29.09 ± 3.70	29.80 ± 3.77	0.3140^b^	–
BMI (kg/m^2^)	22.30 ± 2.77	22.36 ± 2.83	0.3719^a^	−0.0665 (− 0.2142 to 0.0813)
Infertility duration	4.52 ± 2.93	4.58 ± 2.91	0.8809^b^	–
Primary Infert.	70.77% (46/65)	70.77% (46/65)	0.9976^b^	–
Secondary Infert.	29.23% (19/65)	29.23% (46/65)	0.9976^b^	–
Basal E_2_ (pg/mL)	42.92 ± 28.35	42.92 ± 28.43	0.9981^b^	–
Basal FSH (IU/L)	7.27 ± 2.01	7.43 ± 1.94	0.1666^a^	−0.1595 (− 0.3874 to 0.0684)
No. of AFC	13.01 ± 4.15	13.07 ± 3.89	0.5790^a^	−0.1692 (− 0.7758 to 0.4373)

Data are expressed as mean ± SD, and percentage (%). Significant difference; *p* < 0.05; *GnRH-a* Gonadotropin releasing hormone agonist, *CC/Gn* Clomiphene citrate/Gonadotropins, *BMI* Body Mass Index, *E*_*2*_ Estradiol, *FSH* Follicle Stimulating Hormones, *AFC* Anti Follicular Counts, *n* number of patients

^a)^Using Paired t-test; ^b)^ Using Mann-Whitney t-test

**Table 2:** Clinical outcomes

Percentage	Long GnRHa Protocol	CC Protocol	*p-*Values
Implantation rate	–	38.20% (34/89)	–
Clinical pregn/started cycle	9.2% (6/65)	42% (27/65	< 0.0001
Miscarriage rate	–	11.10% (3/27)	–
Cumulative PR/Patients with embryos	14% (6/43)	58% (33/57)	< 0.0001
Cumulative PR/Started cycle	9.2% (6/65)	51% (33/65)	< 0.0001
Av. Number of transferred embryos	1.77 ± 0.43	1.39 ± 0.51	0.0133

Using paired t-test in all. Significant difference; *p* < 0.05

**Table 3:** Stimulation outcomes

Mean ± SD, %		Long GnRHa Protocol	CC Protocol	*p*-Values
Gn total dose (IU)		1719.41 ± 545.199	981.54 ± 236.976	< 0.0001
Gn duration of stimulation (days)		10.14 ± 1.94	9.4 ± 1.27	0.0044
E2 Level at time of hCG trigger (pg/mL)		3077.27 ± 1708.54	3701.96 ± 1805.09	0.012
P Level at time of hCG trigger (ng/mL)		3.96 ± 1.78	4.58 ± 2.91	0.0472
Endometrium thickness (mm)		10.40 ± 2.36	7.36 ± 2.19	< 0.0001
Number of developing follicles		9.26 ± 5.87	9.58 ± 5.65	0.6100
Number of oocytes retrieved		5.98 ± 1.31	7.26 ± 1.95	0.0276
Fertilization rates	IVF	24% (28/115)	64% (87/137)	< 0.0001
	ICSI	30% (11/37)	54% (58/107)	< 0.0001
Number of patients without embryo		33.85% (22/65)	12.31% (8/65)	< 0.0001
Number of patients having frozen embryo		_	81.54% (53/65)	–

Using paired t-test in all. Significant difference; *p* < 0.05. *Gn* Gonadotropin, *E2* Estrogen, *P* Progesterone

## Discussion

Long GnRH agonist protocol is universally regarded as a ‘gold standard’ regimen for controlled ovarian stimulation during IVF treatment of predicted normal responders because of its excellent control of menstrual cycle. GnRH agonist desensitization of its receptors in the pituitary gland by long protocol leads to inhibition of the normal production of endogenous gonadotropins responsible for follicle recruitment, growth, and maturation during the follicular phase [10]. This inhibition on the normal production of endogenous gonadotropins attracts high dose of exogenous gonadotropins. Although it generates more follicles and oocytes, the oocyte quality could be negatively affected. In some cases, there is a high risk of OHSS occurrence and patients ‘drop-out’ phenomenon may increase [10]. Clomiphene citrate stimulates and enhances production of endogenous gonadotropins, hence requires a low dose of exogenous gonadotropins to achieve recruitment, growth, and maturation of follicles. The mimicking of the natural cycle by clomiphene stimulates the process of endogenous gonadotropin (FSH & LH) production [11, 12], and also prevents premature LH surge when administered till maturation triggering [13]. This, therefore, gives clomiphene an advantage over natural cycle and similarity to the long protocol in term of prevention of premature ovulation. Thus, alternative stimulation protocols that can overcome such constraints and still yield same or even better outcomes should be considered.

We evaluated in this study, the efficacy of low dose clomiphene protocol plus low dose gonadotropins in patients with poor IVF results after initial treatment with a long protocol on predicted normal responder patients. The study showed that low dose clomiphene protocol plus low dose gonadotropins were better than long protocol in terms of cumulative pregnancy rate, the number of oocytes retrieved, quality of the embryos generated and the amount of total dose of rFSH used. Thus, clomiphene achieved an optimal result within a shorter time of stimulation, in a low cost-effective and patient-friendly approach. Reports had shown that long GnRH agonist protocol plus higher dosage of gonadotropins appears to affect completion of meiosis resulting in a chromosomal aneuploidy of the oocytes [14], yielding lesser quality embryos after classical ovarian stimulation. This could be a reflection of hypersuperphysiology medication to FSH receptors (FSHR) in the granulosa cells [15, 16] because of the higher dosage and/or prolonged exposure. Other studies had reported that polymorphism of gene could be associated genetically with the use of high dose of gonadotropins [7], which affects signal transduction [17] and inhibit its local vascular network of distribution [18]. However, any of these reasons above could have contributed to the poor response, following long protocol stimulation in the first cycle of this study.

On the other hand, administration of clomiphene citrate till a day before maturation trigger that prevents premature ovulation [13] which is similar to long GnRH agonist protocol does not require a high dose of gonadotropins as needed in long protocol [19]. Furthermore, the poor IVF outcome from the long protocol in this study could not be justified in both total dose of rFSH used and time that will be wasted by the patient, hence the need for change to an alternative protocol such as clomiphene citrate that mimics natural cycle and can also prevent premature ovulation. Also, this alternative was based on low cost, easily accessible, patient-friendly and chances of trying treatments several times in the lesser interval of a period without much delay as in the case of a long protocol. In developing countries such as ours, patients virtually pay all their medical bills. Therefore, in the absence of health insurance coverage on IVF treatment, high cost effective can be checked by the option of using this less expensive and easily accessible protocol, that still give patient good results and save time for more chances of trying severally in a short interval.

Clomiphene plus a low dose of rFSH regimen can also overcome single follicular dominance and promote multiple follicular growths [20, 21]. A positive IVF result decreases patient’s anxiety, frustration and rate of discontinuation of treatment; and this has been reported by researchers to be associated with mild stimulation [22]. The mimicking nature of clomiphene citrate may have contributed to the retrieved quality oocytes [12], as there is good bidirectional signaling between oocytes and granulosa cells essential for follicular recruitment, development, growth, and acquisition of oocyte competence with natural maturation of nucleus and cytoplasm [23, 24]. Thus, there were better fertilization rate and high-quality embryos [25] in CC based protocol than in the long protocol of this study. There are some limitations in clomiphene protocol with respect to the thin endometrium and freeze all embryos phenomenon. However, the time interval between frozen embryo transfers could sometimes be shorter when compared to the interval taken by the long protocol that might still fail. A natural cycle prepared endometrium protocol for frozen-thawed embryo transfer could also attract follicle growth and oocyte retrieval, thereby generating more embryos.

Another limitation of this study was the tendency of regression closer to the mean that is usually seen in retrospective studies of poor responders. However a previous study reported in detail, a cumulative pregnancy rate for five cycles with a constant pregnancy rate and the success rate only started declining from the sixth cycles [26]. In their study, fertilization was referred to either regular IVF or ICSI insemination, and also demonstrated that a number of patient characteristics, particularly their age can diminish the rate of pregnancy after IVF cycles [27]. Patients older than thirty years have a significantly lower pregnancy rate in IVF/ICSI cycles. Although there was relatively small sample described in this study, the pregnancy rate was statistical significance in the IVF cycle using clomiphene protocol. Multiple analyses were performed for all the variables and all the data simultaneously.

Furthermore, the present study demonstrated that in addition to the good pregnancy rate, the number of eggs fertilized and fertilization rate could be important factors in determining the outcome, despite the low average number of transferred embryos in the second cycle. Patients with a fertilization rate lower than 45% needed statistically significantly more cycles to achieve a pregnancy. This low fertilization rate could possibly be as a result of poor quality oocytes and/or poor quality sperm. Many studies have explained such poor fertilization after IVF/ICSI cycles to be due to the oocyte quality and/or sperm quality [28–32].

## Conclusion

In conclusion, this study demonstrated that low dose clomiphene protocol was better than long GnRH agonist protocol in terms of cumulative pregnancy rate, the number of oocytes retrieved and lesser total dose of recombinant FSH used. Thus, clomiphene treatment can be an alternative option to such patients in second cycle stimulation instead of repeating long protocol regimen. There is a need for randomized controlled studies with larger number of patients.
